# Coexistence of hereditary transthyretin amyloid cardiomyopathy and sarcomeric hypertrophic cardiomyopathy: a multimodality imaging and genetic case report

**DOI:** 10.1093/ehjcr/ytag515

**Published:** 2026-07-10

**Authors:** Francesco Negri, Mauro Driussi, Michela Puppato, Lorenzo Zuliani, Massimo Imazio

**Affiliations:** Cardiothoracic Department, University Hospital ‘Santa Maria della Misericordia’, Azienda Sanitaria Universitaria Friuli Centrale (ASUFC), Piazzale Santa Maria della Misericordia 15, 33100 Udine, Italy; Cardiothoracic Department, University Hospital ‘Santa Maria della Misericordia’, Azienda Sanitaria Universitaria Friuli Centrale (ASUFC), Piazzale Santa Maria della Misericordia 15, 33100 Udine, Italy; Radiology Department, University Hospital ‘Santa Maria della Misericordia’, Azienda Sanitaria Universitaria Friuli Centrale (ASUFC), Piazzale Santa Maria della Misericordia 15, 33100 Udine, Italy; Radiology Department, University Hospital ‘Santa Maria della Misericordia’, Azienda Sanitaria Universitaria Friuli Centrale (ASUFC), Piazzale Santa Maria della Misericordia 15, 33100 Udine, Italy; Cardiothoracic Department, University Hospital ‘Santa Maria della Misericordia’, Azienda Sanitaria Universitaria Friuli Centrale (ASUFC), Piazzale Santa Maria della Misericordia 15, 33100 Udine, Italy; Department of Medicine, University of Udine, 33100 Udine, Italy

**Keywords:** Transthyretin amyloidosis, Hypertrophic cardiomyopathy, MYH7, TTRv p.Ile88Leu, Multimodality imaging, Genetic testing, Case report

## Abstract

**Background:**

Unexplained left ventricular hypertrophy (LVH) represents a diagnostic challenge, as phenotypic overlap may exist between sarcomeric hypertrophic cardiomyopathy (HCM) and infiltrative cardiomyopathies such as transthyretin amyloid cardiomyopathy (ATTR-CM). Multimodality imaging and genetic testing are crucial when standard findings are discordant.

**Case summary:**

A 78-year-old woman presented with exertional dyspnoea (NYHA class II) and asymmetric septal hypertrophy without secondary causes. Transthoracic echocardiography showed preserved left ventricular ejection fraction with reduced basal longitudinal strain and relative apical sparing. Cardiac magnetic resonance demonstrated elevated native T1 values (1240–1270 ms), mildly increased T2 values, and basal ring-like late gadolinium enhancement. Bone scintigraphy revealed Perugini grade 3 myocardial uptake, consistent with ATTR-CM. Monoclonal gammopathy was excluded. Genetic testing identified a pathogenic transthyretin mutation (TTR p.Ile88Leu) and a concomitant likely pathogenic sarcomeric MYH7 variant (p.Gly733Arg). Neurological assessment was unremarkable. The overall phenotype was dominated by amyloid infiltration.

**Discussion:**

Following multidisciplinary evaluation, the patient was started on tafamidis 61 mg once daily. Cascade genetic and clinical screening was recommended for first-degree relatives.

Learning points

- This case highlights how dual cardiomyopathy substrates may alter conventional imaging signatures of ATTR-CM and complicate phenotype-specific therapeutic decision-making.
- Comprehensive genetic testing in unexplained LVH enables identification of dual hereditary substrates, guiding disease-modifying therapy and mandating cascade screening of first-degree relatives for both conditions.

## Introduction

Left ventricular hypertrophy is commonly attributed to arterial hypertension, aortic stenosis, or sarcomeric hypertrophic cardiomyopathy.^1^ However, infiltrative diseases such as transthyretin amyloid cardiomyopathy (ATTR-CM) may mimic or overlap with these conditions. Advances in deformation strain imaging, cardiac magnetic resonance (CMR), and bone scintigraphy have improved diagnostic accuracy, particularly in patients with atypical or discordant findings.^2–7^ We report a case of unexplained LVH in which multimodality imaging and genetic testing revealed a rare coexistence of hereditary ATTR-CM and a sarcomeric MYH7 variant.

## Summary figure

**Figure:**
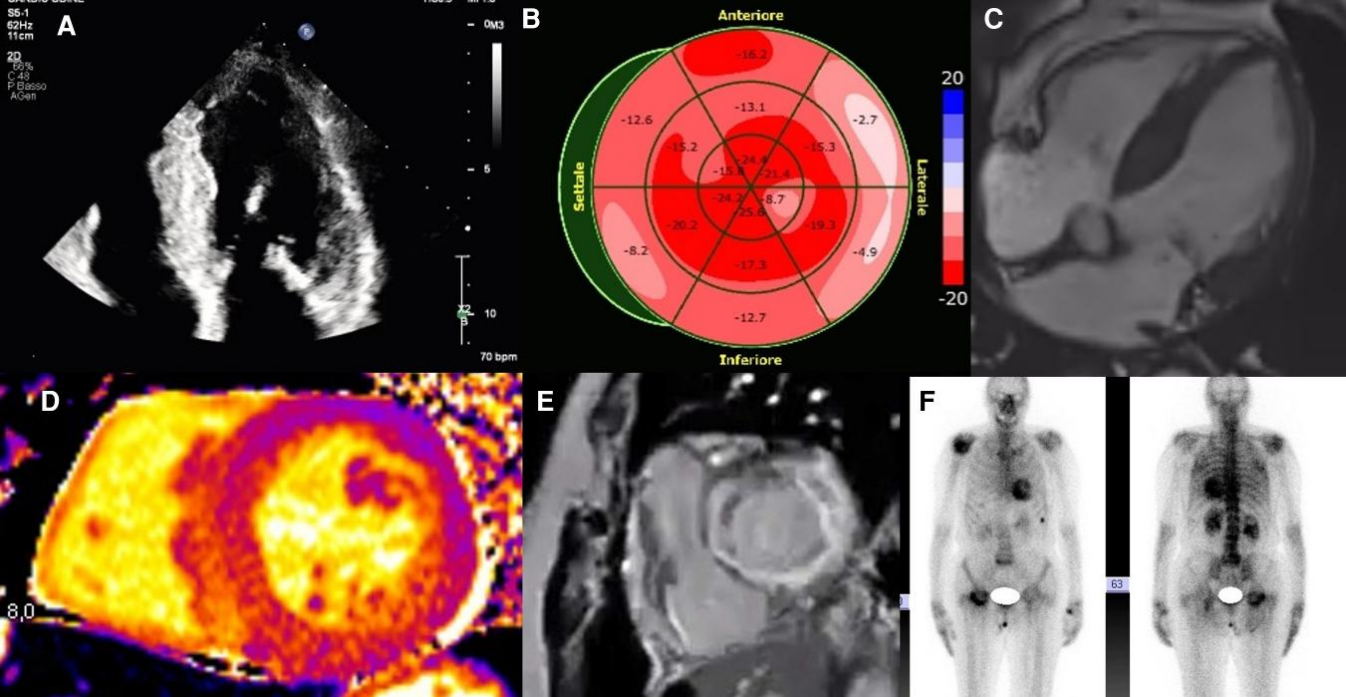


## Case presentation

A 78-year-old woman was referred for evaluation of exertional dyspnoea (NYHA class II).

She denied chest pain, palpitations, syncope, or heart failure symptoms at rest.

Her medical history included rheumatoid arthritis, well controlled with abatacept, and hypercholesterolaemia. She had no history of arterial hypertension, diabetes mellitus, smoking, or coronary artery disease. Family history was notable for maternal death due to heart failure at 73 years of age, with unknown aetiology.

Physical examination was unremarkable. Baseline ECG showed no evidence of LVH or low QRS voltages. Transthoracic echocardiography demonstrated normal left ventricular dimensions with asymmetric septal hypertrophy with a maximum thickness of 15 mm (Panel *A*) and preserved systolic function (LVEF 68%). Global longitudinal strain analysis revealed reduced strain in basal segments with relative apical sparing (Panel *B*). Diastolic function assessment was consistent with impaired relaxation (E/A < 1), with non-diagnostic filling pressure estimates (E/e′ 13). Right ventricular size and function were normal.

Given the absence of secondary causes and discordant ECG–echocardiographic findings, further evaluation was pursued. CMR confirmed asymmetric septal hypertrophy (Panel *C*) and revealed markedly elevated basal native T1 values (1240–1270 ms; Panel *D*) with mildly increased T2 values. Late gadolinium enhancement showed a basal ring-like intramyocardial and partially transmural pattern (Panel *E*). Although classic CMR features of amyloidosis were incomplete (myocardial nulling was normal and there was no early darkening of the myocardium), the overall findings suggested an infiltrative process.

Bone scintigraphy demonstrated Perugini grade 3 myocardial uptake, supporting a diagnosis of ATTR-CM^3,8^ (Panel *F*). Serum and urine studies excluded monoclonal gammopathy. Genetic testing for hypertrofic phenotype identified a heterozygous pathogenic TTR variant (c.262A > T; p.Ile88Leu) and a heterozygous likely pathogenic MYH7 variant (c.2197G > A; p.Gly733Arg).^9^ NT-proBNP was mildly elevated (424 ng/L), and high-sensitivity troponin was within normal limits eGFR 50 ml/min; NAC staging I.

Neurological assessment revealed no evidence of peripheral or autonomic neuropathy. Exercise testing and 24-hour Holter monitoring showed no clinically relevant arrhythmias. The phenotype was considered predominantly driven by amyloid infiltration.

Following multidisciplinary evaluation, the patient was started on tafamidis 61 mg once daily, in accordance with current guideline recommendations for symptomatic ATTR-CM with NYHA Class II symptoms and no neurological involvement.^10^ The concomitant MYH7 p.Gly733Arg variant was acknowledged; however, given the predominance of the amyloid phenotype and the absence of haemodynamically significant left ventricular outflow tract obstruction, specific HCM-directed pharmacological therapy was not initiated at that time. Given the emerging evidence supporting the use of SGLT2 inhibitors in patients with ATTR-CM, an SGLT2 inhibitor was also initiated.^11^

The patient was enrolled in a structured follow-up programme including serial echocardiography, NT-proBNP, high-sensitivity troponin, and 6-min walk test. Cascade genetic and clinical screening was formally recommended for all first-degree relatives, in accordance with guidelines for hereditary cardiomyopathies.

## Discussion

The TTR p.Ile88Leu variant—endemic in Italy and Southern Europe and associated with a predominantly cardiac phenotype—closely aligned with the patient’s age at onset and clinical presentation. Although traditionally labelled as ‘cardiac predominant’, recent studies suggest that up to two-thirds of affected individuals may demonstrate neurological involvement when systematically assessed.^12^ The absence of neuropathy in our patient is therefore consistent with the reported phenotypic spectrum but warrants periodic neurological reassessment.

The atypical CMR presentation in this patient with markedly elevated native T1 but preserved myocardial nulling and absent diffuse subendocardial LGE warrants discussion. In isolated ATTR-CM, diffuse subendocardial or transmural LGE and early myocardial gadolinium uptake are hallmarks of significant amyloid burden. In this case, the concomitant presence of the MYH7 p.Gly733Arg variant associated with myocyte disarray, focal fibrosis, and altered extracellular matrix composition may have contributed to a modified LGE pattern. Specifically, sarcomeric HCM can produce a focal midwall or basal ring-like LGE pattern that could partially mask or distort the diffuse subendocardial pattern typically seen in amyloidosis. Furthermore, relatively early or moderate amyloid burden (supported by the relatively preserved EF and mildly elevated NT-proBNP) may not have reached the threshold required for classic LGE and nulling reversal. The elevated T1 values, which directly reflect myocardial interstitial expansion, may therefore be the earliest and most sensitive indicator of amyloid infiltration in this dual-pathology context.^6, 7^

The coexistence of ATTR-CM and sarcomeric HCM creates unique therapeutic challenges.

TTR-directed disease-modifying therapy currently tafamidis or acoramidis (TTR stabilizers), or vutrisiran (RNA silencer) should be prioritized when ATTR-CM is the dominant phenotype, as in the present case. Tafamidis was initiated in accordance with recommendations for symptomatic ATTR-CM without neurological involvement.^10^ Beta-blockers are Class I recommended therapy in obstructive HCM,^1^ but in ATTR-CM their use may precipitate hypotension or fatigue due to chronotropic dependence. Furthermore, a recently published small, randomized trial showed that the β1-selective beta-blocker bisoprolol reduced exercise capacity and worsened several diastolic and biomarker parameters in non-obstructive HCM.^13^

Cardiac myosin inhibitors (mavacamten, aficamten) were considered inappropriate given the absence of obstruction and the lack of safety data in amyloid infiltration. In addition to tafamidis 61 mg, we decided to initiate an SGLT2 inhibitor, since emerging data in ATTR-CM^11^ suggests that these agents may represent a rational adjunctive therapy in mixed phenotypes, pending definitive trial data.

Given the autosomal dominant nature of both identified variants, cascade genetic testing was recommended for all first-degree relatives, namely her 55-year-old son and her 71-year-old sister, in accordance with established guidelines for hereditary cardiomyopathies. Familial screening results were pending at the time of this report. This underscores the critical importance of genetic counselling infrastructure in centres managing patients with hereditary cardiomyopathies.

Previous studies have reported overlaps between HCM phenotypes and ATTR. Kanelidis *et al*.^14^ described pathogenic TTR variants in patients with phenotypic HCM, emphasizing that this association is likely underrecognized and that affected patients require tailored diagnostic evaluation and longitudinal surveillance for ATTR deposition. Cappelli *et al*.^15^ showed that tenosynovial manifestations, particularly carpal tunnel syndrome and biceps tendon rupture, may help identify ATTR-CM among patients with an HCM phenotype. Our case extends these observations by documenting a true dual genetic substrate, with confirmed hereditary ATTR-CM due to the TTR p.Ile88Leu variant coexisting with a likely pathogenic MYH7 p.Gly733Arg variant. In contrast to previous reports focused mainly on recognition and screening, the present case highlights how overlapping cardiomyopathy substrates may modify conventional imaging signatures, complicate phenotype attribution, and directly influence therapeutic decision-making and cascade family screening.

## Patient’s perspective

I initially experienced shortness of breath without understanding the cause. The diagnostic process helped clarify my condition and available treatments, and I was informed that this information may also be important for my family.

## Conclusion

This case demonstrates that hereditary ATTR-CM may coexist with sarcomeric cardiomyopathy variants, creating overlapping phenotypes that can modify conventional imaging signatures and complicate phenotype attribution. Recognition of dual genetic cardiomyopathy substrates has important implications for personalized therapeutic decision-making, longitudinal follow-up, and cascade family screening, reinforcing the central role of multidisciplinary evaluation and genetic testing in the diagnostic work-up of unexplained LVH.

## Data Availability

The data underlying this article are available within the article and its supplementary materials.

## Lead author biography

Francesco Negri, MD is a clinical cardiologist at the Cardiothoracic Department of the University Hospital ‘Santa Maria della Misericordia’, Azienda Sanitaria Universitaria Friuli Centrale (ASUFC), Udine, Italy, with clinical and research interests in cardiomyopathies, multimodality cardiac imaging, especially, CMR, and inherited cardiovascular diseases.

## Notes

**Consent:** Written informed consent was obtained from the patient for publication of this case report and any accompanying images. This case report has been prepared in accordance with the CARE guidelines.
